# Total Dystrophic Onychomycosis of All the Nails Caused by Non-dermatophyte Fungal Species: A Case Report

**DOI:** 10.7759/cureus.29765

**Published:** 2022-09-29

**Authors:** Settipally Nikitha, Neha Kondraganti, Venkataramana Kandi

**Affiliations:** 1 Microbiology/Internal Medicine, Prathima Institute of Medical Sciences, Karimnagar, IND; 2 Internal Medicine, Prathima Institute of Medical Sciences, Karimnagar, IND; 3 Clinical Microbiology, Prathima Institute of Medical Sciences, Karimnagar, IND

**Keywords:** cladosporium spp, fungal infection, fingernails, toenails, nail biopsy, molds, non-dermatophytes, nail clippings, total dystrophic onychomycosis, onychomycosis

## Abstract

Onychomycosis (OM) is a common disease of nails. It is a major public health issue worldwide due to its increasing prevalence. Moreover, OM is associated with substantial consequences including physical and psychological outcomes. Dermatophytes are a group of keratinophilic fungi that account for more than half of OM incidences. However, yeasts and non-dermatophyte molds represent about one-third of OM infections that have recently shown an increasing trend. The knowledge of OM appears to be low among people, especially those who live in rural areas. Therefore, most cases remain undiagnosed, which in turn leads to the spread of fungus within the individual and to other susceptible people. We report a case of total dystrophic onychomycosis involving all the fingernails and toenails in an 87-year-old female patient caused by non-dermatophyte molds.

## Introduction

The name onychomycosis (OM) is derived from the Greek word 'onyx' meaning nail and 'mykes' meaning fungus. OM is the clinical terminology used to refer to the fungal infection of the nail. The majority of OM cases involve infection by dermatophyte group of fungi and when nail infection is caused by such fungi, the clinical condition is termed tinea unguium. The infection may involve the fingernails as well as toenails; however, the latter is more commonly affected. OM may present as distal subungual OM (DSO), distal and lateral subungual OM (DLSO), proximal subungual OM (PSO), white superficial OM (WSO), and total dystrophic OM (TDO) [1,2]. The etiology of OM includes dermatophytes, yeasts (*Candida*), and non-dermatophyte molds (NDM). The infected nails show thickening, discoloration, and separation from the nail bed. The incidence of OM is 10% among the general population, 20% in persons older than 60 years, and 50% among those older than 70 years of age. The majority of OM cases are attributed to infection with anthropophilic dermatophytes that include *Trichophyton rubrum*, *Trichophyton mentagrophytes*, and *Epidermophyton floccosum*, among others [1].

OM caused by NDM is less common among healthy populations. NDM, like *Scopulariopsis brevicaulis *and *Aspergillus *species (spp.), have been reported to cause OM either exclusively or in mixed infections along with dermatophytes [3]. Other NDM that have been isolated from OM cases include *Fusarium *spp., *Acremonium *spp., *Alternaria *spp., Scytalidium spp., and *Neoscytalidium *spp., among others.

OM of the fingernails or toenails may lead to pain, discomfort, and impaired tactile functions. It may also interfere with walking, exercise, or proper shoe fit. Fungal infection of the nails may predispose people to secondary bacterial infections, cellulitis, idiopathic reactions, and chronic urticaria. In addition, OM causes both psychosocial and physical morbidity that greatly affects the quality of life [4]. We report a rare case of total dystrophic OM of both fingernails and toenails in an 87-year-old female patient caused by NDM.

## Case presentation

**Figure 1:**
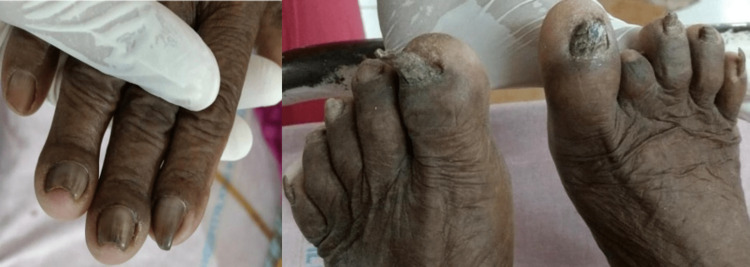
Total dystrophic onychomycosis of finger and toe nails

An 87-year-old female patient was referred to the Department of Dermatology attached to Prathima Institute of Medical Sciences, Karimnagar, Telangana, India, for evaluation of nail infection. She had a significant past history of hypertension and was admitted to the medical ward of the same hospital for the management of severe and uncontrolled hypertension. There was no history of trauma, diabetes mellitus, or any other systemic disease at the time of presentation. She was on anti-hypertensive medication for many years. However, the patient gave a history of working with tobacco leaves with bare hands, which was a part of her occupation. She gave a history of deformation of nails since childhood. The changes were reported to be first seen in the toenails, which gradually spread to the fingers. No interference/discomfort with standing and walking was reported by the patient. No history of paresthesia, distress, pain, itching, or loss of dexterity was reported. The patient gave no previous history of respiratory infections and allergies. A family history of similar nail changes was present. The patient was well built; however, at presentation was unconscious and disorientated to time, place, and persons. On general examination, we observed grossly deformed nails of all 10 digits as shown in Figure 1.

**Figure 2:**
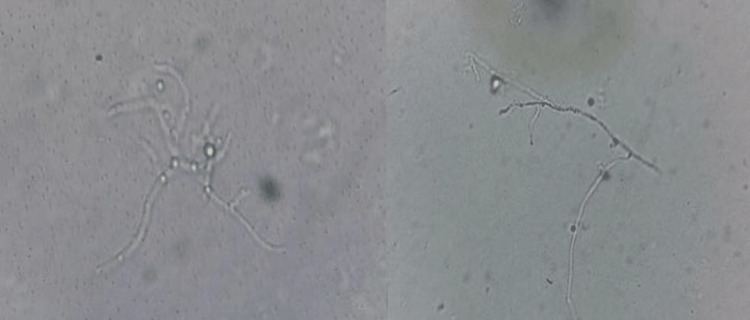
Potassium hydroxide mount of the nails showing the presence of fungal hyphae/mycelia

The nails were thickened and hardened, showing loss of texture and brownish-black discoloration. No signs of inflammation and pain in the peri-ungual region were observed. Nail clippings and scrapings were collected and processed using 20-40% potassium hydroxide (KOH). Microscopic examinations of the KOH mount revealed two types of fungal hyphae/mycelia as shown in Figure 2.

**Figure 3:**
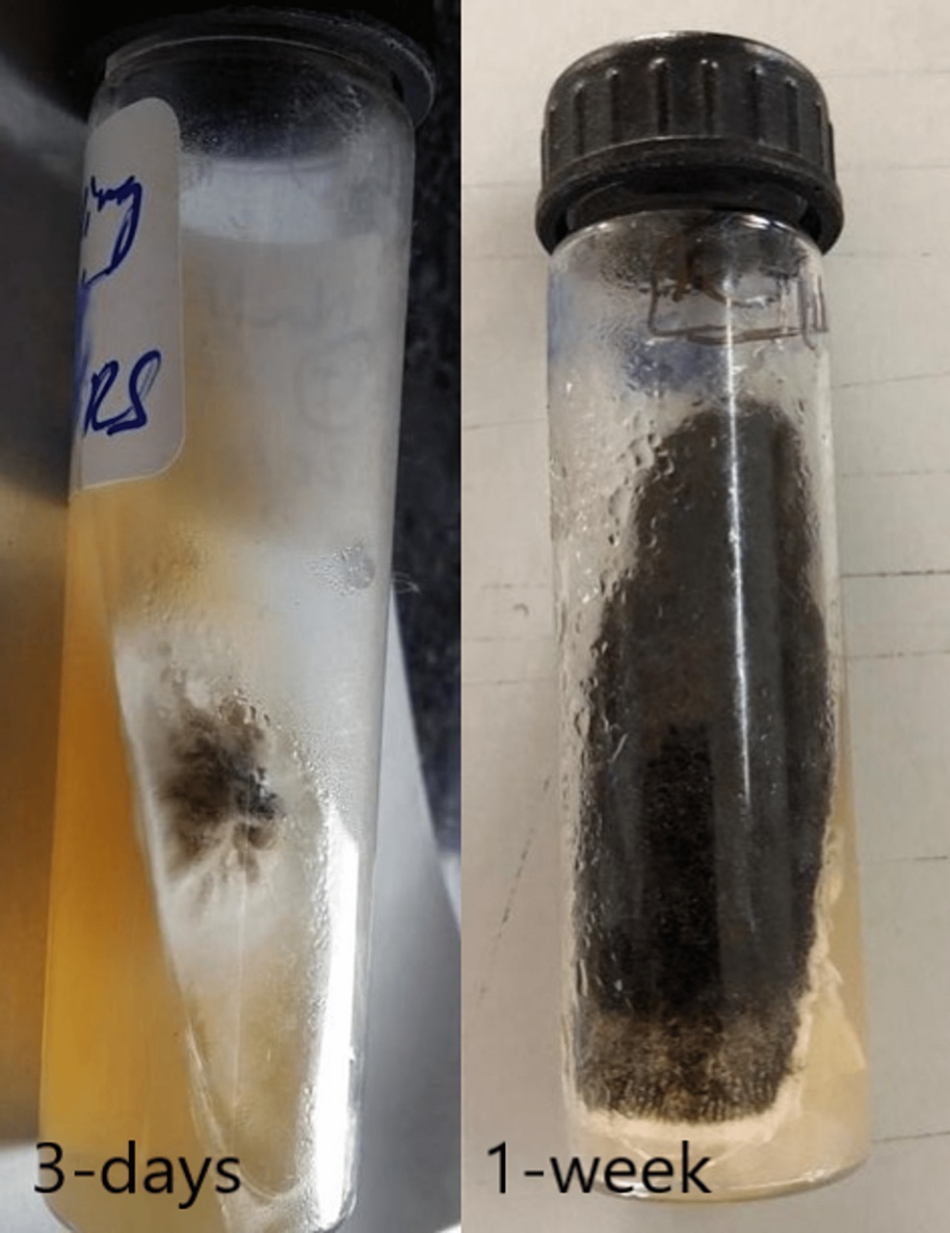
The Sabouraud dextrose agar culture incubated at room temperature shows the growth of mold

The nail samples were cultured on Sabouraud dextrose agar (SDA), where one tube was incubated at room temperature (20-30^0^C) and the other at 37^0^C. The tube incubated at room temperature showed the growth of a greyish, wooly colony on the third day, which on further incubation for a week, turned velvety black. The reverse of the velvety black colony showed brownish-black discoloration as shown in Figure 3.

**Figure 4:**
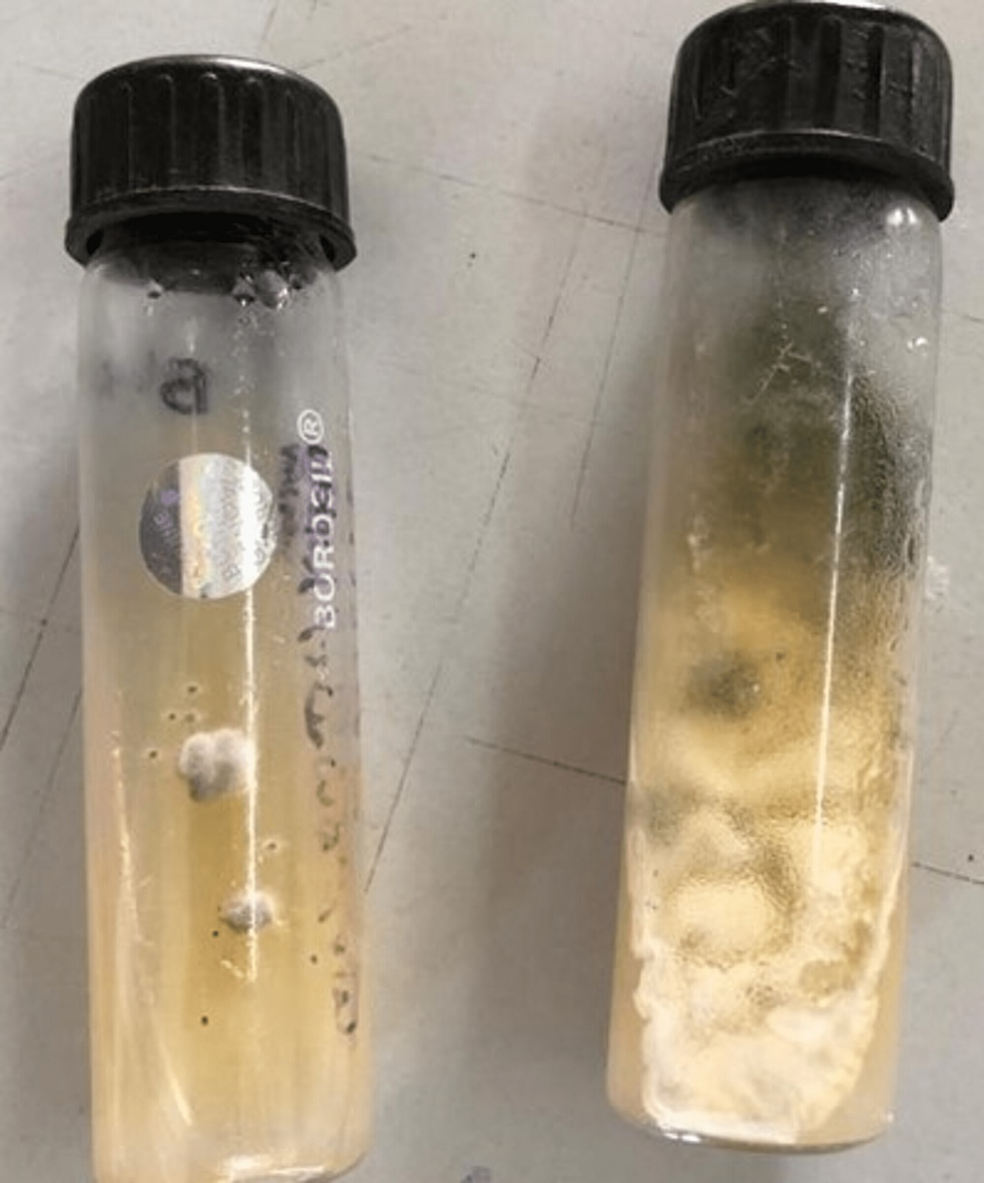
The Sabouraud dextrose agar culture from the incubator shows white cloudy textured mold growth

Tube incubated at 37^0^C showed white cloudy colonies as shown in Figure 4.

**Figure 5:**
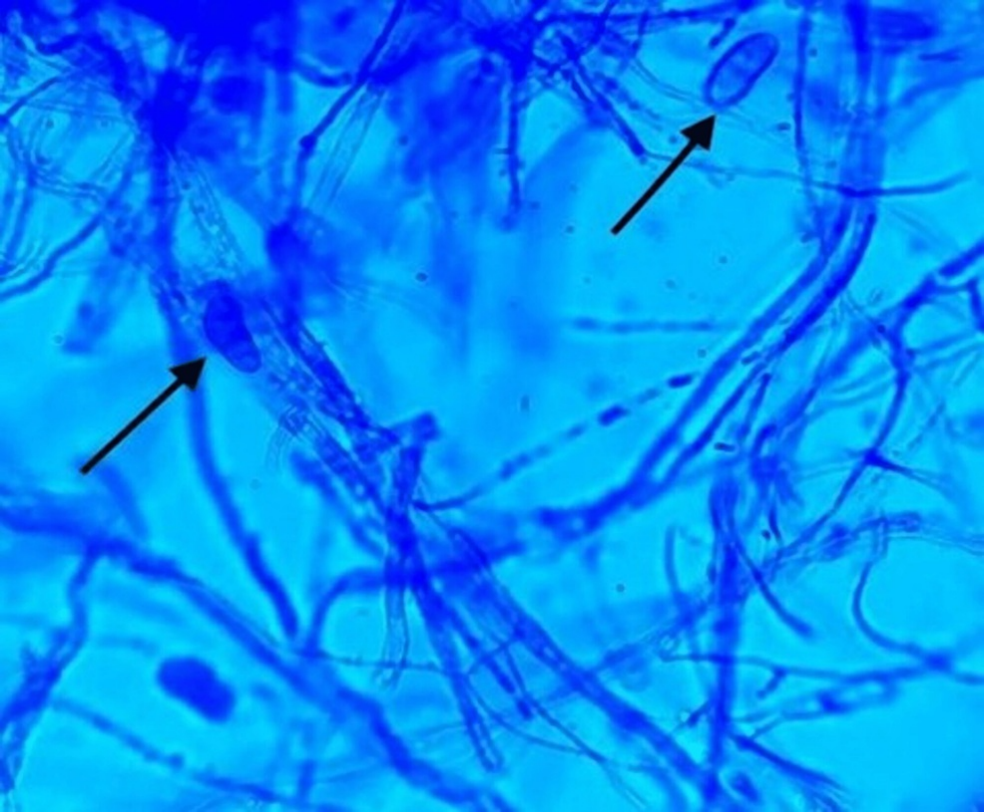
The lactophenol cotton blue mount of the velvety black colonies shows the presence of hyphae and conidia (black arrows)

The lactophenol cotton blue (LPCB) mount of the velvety black colony showed the presence of conidia (fruiting bodies) as shown in Figure 5.

**Figure 6:**
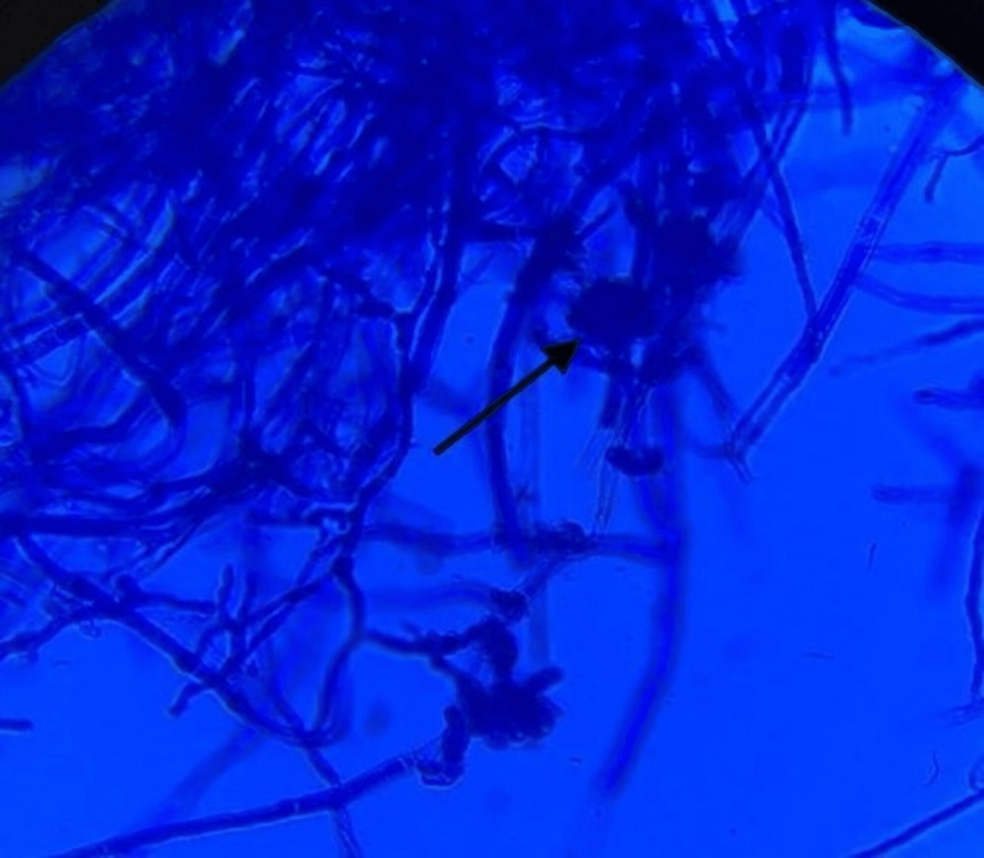
The lactophenol cotton blue mount of white cloudy colonies shows hyphae and fruiting bodies (black arrow)

The white cloudy colonies on the LPCB mount revealed the presence of hyphae along with fruiting bodies as shown in Figure 6.

The fungus showing velvety black pigment on SDA revealed hyphae that were branched, septate, and pale brown in color on the LPCB mount. The conidia were cylindrical/globose and septate. It was morphologically identified as *Cladosporium *spp. The white cloudy colonies on SDA revealed hyphae which were branched, septate, and dark brown in color on the LPCB mount. The conidia were septate and appeared dark brown in color. Based on these observations, it was identified as *Curvularia *spp. Due to the unavailability of advanced/molecular methodologies, the fungal species could not be confirmed. Ideally, the patient should be prescribed oral terbinafine, fluconazole, or itraconazole 250 milligrams once a day for 12 weeks and continually followed up during the treatment. Other modalities include palliative treatment with mechanical or chemical debridement, topical anti-fungal therapy, or a combination of these modalities. Considering the age, present clinical condition, and potential side effects of the antifungal therapy, no treatment was initiated in our patient.

## Discussion

OM is a highly prevalent infection worldwide that affects 5-10% of the world's population. More than 60% of OM cases are attributed to dermatophytes and up to 40% of OM cases may involve mixed infections with dermatophytes and NDM [5]. NDM causes OM of toenails in elderly individuals with a history of trauma and has been recently showing an increasing trend [6]. According to the available evidence, they have become the main causative organism in patients with OM and HIV infection. These fungi can penetrate the nail via the distal subungual region and the lateral nail track, through the dorsal surface of the nail plating to causing superficial onychomycosis, or through the under-surface of the proximal fold of the nail [7].

The prevalence of onychomycosis is particularly high in warm humid climates. People living in certain habitats, those who have the habit of walking barefoot, wearing ill-fitting shoes, nail-biting (onychophagia), or with a working environment that exposes them to wetness and chemicals could predispose them to OM. The prevalence of OM raises with age, perhaps due to inadequate peripheral circulation, diabetes mellitus, repeated nail trauma, protracted exposure to pathogenic fungi, suboptimal immune function, inactivity, or inability to trim the toenails and care for the feet. Women involved in household work like cutting and peeling vegetables, washing utensils, cleaning the house/laundry, and those who are chronically exposed to moist environments are predisposed to fungal infections [8,9].

In geriatric/aged people, there is an increased occurrence of OM associated with inferior treatment responses. Toenails are 25 times more likely to be infected than fingernails as the causative molds are ubiquitous fungi present in the soil, water, and decaying vegetation. Infected nails may serve as a reservoir for fungi, enabling their transmission to other areas of the body and person-person transmission [10].

There is a diverse array of therapies for treating OM, particularly centered on topical formulas as the adverse effects are limited to the application site without systemic drug interactions. Newer diagnostic techniques like conventional polymerase chain reaction (PCR), real-time PCR techniques, and matrix-assisted laser desorption ionization-time of flight mass spectrometry (MALDI-TOF MS) are now dependable for performing the in-situ identification of dermatophytes, yeasts, and NDMs in OM provided that adequate nail material is collected. Non-invasive methods such as optical coherence tomography, confocal laser scan, and advanced microscopic methods including phase contrast microscopy and hard X-ray microscopy, help in early diagnosis and better management of infection [11].

## Conclusions

In the present case, neither the 87-year-old patient nor her family members were aware of the infection and, instead, they had the perception that this change was normal and age-related. This led to the spread of the infection to the nails of all 10 digits. Despite the diagnosis, treatment could not be initiated in this patient due to the extreme age and potential adverse effects of antifungal drugs. Although not life-threatening, it is important that patients and healthcare professionals don’t underestimate the condition. If left untreated, the fungus may disseminate and persist, resulting in substantial health complications. We suggest the need for careful mycological examination and appropriate medical intervention at earlier stages of infection. Therefore, interpersonal communication regarding personal hygiene should be encouraged. The government and public health professionals should take initiatives to spread awareness about fungal infections amongst people, especially in rural areas.
